# BCAT1 Activates PI3K/AKT/mTOR Pathway and Contributes to the Angiogenesis and Tumorigenicity of Gastric Cancer

**DOI:** 10.3389/fcell.2021.659260

**Published:** 2021-06-07

**Authors:** Xiong Shu, Pan-Pan Zhan, Li-Xin Sun, Long Yu, Jun Liu, Li-Chao Sun, Zhi-Hua Yang, Yu-Liang Ran, Yue-Min Sun

**Affiliations:** ^1^Laboratory of Molecular Orthopedics, Beijing Jishuitan Hospital, Beijing Research Institute of Orthopedics and Traumatology, Beijing, China; ^2^State Key Laboratory of Molecular Oncology, National Cancer Center/National Clinical Research Center for Cancer/Cancer Hospital, Chinese Academy of Medical Sciences and Peking Union Medical College, Beijing, China; ^3^Department of Pancreatic and Gastric Surgery, National Cancer Center/National Clinical Research Center for Cancer/Cancer Hospital, Chinese Academy of Medical Sciences and Peking Union Medical College, Beijing, China

**Keywords:** branched-chain aminotransferases, BCAT1, angiopoiesis, tumorigenicity, oncogene, gastric cancer

## Abstract

**Background:**

Focusing on antiangiogenesis may provide promising choices for treatment of gastric cancer (GC). This study aimed to investigate the mechanistic role of BCAT1 in the pathogenesis of GC, particularly in angiogenesis.

**Methods:**

Bioinformatics and clinical samples analysis were used to investigate the expression and potential mechanism of BCAT1 in GC. BGC823 cells with BCAT1 overexpression or silencing were induced by lentiviral transduction. Cell phenotypes and angiogenesis were evaluated. The relevant proteins were quantized by Western blotting, immunohistochemistry, or immunofluorescence. Xenograft models were constructed to confirm the role of BCAT1 *in vivo*.

**Results:**

BCAT1 was overexpressed in GC patients and associated with lower survival. BCAT1 expression was correlated with proliferation-, invasion-, or angiogenesis-related markers expression and pathways. Silencing BCAT1 expression suppressed cell viability, colony formation, cycle progression, invasion, and angiogenesis of BGC823 cells, as well as the tumor growth of xenograft models, whereas overexpressing BCAT1 had the opposite results both *in vitro* and *in vivo*. Bioinformatics analysis and Western blotting demonstrated that BCAT1 activated the PI3K/AKT/mTOR pathway. The addition of LY294002 reversed the tumor growth induced by BCAT1 overexpression, further verifying this mechanism.

**Conclusion:**

BCAT1 might act as an oncogene by facilitating proliferation, invasion, and angiogenesis through activation of the PI3K/AKT/mTOR pathway. This finding could aid the optimization of antiangiogenesis strategies.

## Introduction

Gastric cancer (GC) is the third leading cause of cancer-related deaths worldwide (Venerito et al., 2018). Despite a declining incidence over recent decades, GC remains a major killer across the globe (Karimi et al., 2014). GC has a high mortality rate, as it is often diagnosed at an advanced stage and treatment options are limited (Necula et al., 2019). Surgery remains the only curative treatment; however, most patients have inoperable disease at diagnosis or develop a relapse after resection with curative intent (Van Cutsem et al., 2016). For patients with advanced disease, systemic chemotherapy, including adjuvant or neoadjuvant chemotherapy, remains the mainstay of treatment (Wagner et al., 2017). Several novel drugs targeting dysfunctional molecular pathways have been developed for GC treatment, although targeted therapies are currently limited to HER-2–positive disease (Cervantes et al., 2013; Lazãr et al., 2016). The optimal choice of regimen is still uncertain. Thus, it is essential to identify specific molecular patterns and biomarkers that could be used to develop therapies targeted to individual tumor behaviors.

Tumor angiogenesis is an essential process for the growth, metastasis, and progression of solid tumors, supplying rapidly growing malignant tissues with essential nutrients and oxygen (Kzhyshkowska et al., 2014). Thus, controlling tumor angiogenesis is a promising therapeutic strategy for limiting tumor progression (Weis and Cheresh, 2011). A wide evidence demonstrates that GC is a highly angiogenic cancer (Chakroborty et al., 2004; Capuano et al., 2019); thus, focusing on antiangiogenesis may provide promising choices for GC treatment.

Branched-chain amino acid transaminase 1 (BCAT1) is the predominant isoform of BCAT that initiates the catabolism of branched-chain amino acids (BCAAs) (Papathanassiu et al., 2017). The activity of BCAT in tumor growth has recently aroused considerable interest. Tönjes et al. (2013) reported high levels of BCAT1 in gliomas, and several subsequent studies demonstrated the central role of BCAT1 in tumor pathogenesis, including in breast cancer (Thewes et al., 2017), leukemia (Raffel et al., 2017), ovarian cancer (Wang et al., 2015), and nasopharyngeal carcinoma (Zhou et al., 2013). Notably, gliomas and ovarian cancer are also highly angiogenic tumors (Farhadi et al., 2005; Campos et al., 2007); accordingly, we assumed that BCAT1 might act as a tumor oncogenic factor through controlling tumor angiogenesis. However, the mechanistic role of BCAT1 in the pathogenesis of GC, particularly in angiogenesis, remains largely uncertain.

In the present study, we verified that BCAT1 was overexpressed in GC patients with poor prognosis through analyzing data from The Cancer Genome Atlas (TCGA) and clinical tumor samples. Our findings indicated that BCAT1 promoted proliferation, invasion, and angiogenesis by activating the PI3K/AKT/mTOR pathway, thereby functioning as an oncogenic factor both *in vitro* and *in vivo*. Thus, our data suggest that BCAT1 may be an effective target for GC treatment.

## Materials and Methods

### Bioinformatics Analysis

RNA sequencing read counts for GC samples were downloaded from the TCGA portal^1^ for the analysis of BCAT1 expression. The limma package of the R software was used to transform the raw microarray data into mRNA expressions. Log-rank test with Kaplan–Meier survival curves was performed to compare overall survival (OS) between patients with high and low BCAT1 expression. Time receiver operating characteristic (ROC) curve analysis was performed to evaluate the predictive accuracy of BCAT1. Differentially expressed genes (DEGs) were screened using thresholds of adjusted *p* < 0.05 and | log (fold change)| > 1. To further analyze the underlying function of potential targets, Gene Ontology (GO) analysis, Kyoto Encyclopedia of Genes and Genomes (KEGG) pathway enrichment analysis, and Gene Set Enrichment Analysis (GSEA) were performed using the DAVID online tools. Enriched pathways were identified using thresholds of adjusted *p* < 0.05 and false discovery rate <0.25.

### Clinical Samples

Paraffin-embedded GC tissues (*n* = 93) and paracarcinoma tissues (*n* = 8) were obtained from the Cancer Hospital, Chinese Academy of Medical Sciences (Beijing, China). Samples were collected from patients undergoing surgical resection. All patients were histologically diagnosed and had no history of other malignancies or previous chemoradiotherapy. Informed consent was obtained from each patient, and the protocol was approved by the Institutional Review Committee of the Cancer Hospital, Chinese Academy of Medical Sciences.

### Immunohistochemical Staining

Immunohistochemical (IHC) staining for BCAT1, p-AKT, CD31, vascular endothelial growth factor (VEGF), Ki67, and CD44 expression was performed using an SP-IHC kit (Zhongshan Jinqiao Company, Beijing, China) according to the standard protocol. Paraffin sections were deparaffinized, rehydrated, and treated with sodium citrate buffer for antigen retrieval. After blocking endogenous peroxidase activity and non-specific staining, sections were subsequently incubated with primary and secondary antibodies. Next, sections were incubated with diaminobenzidine (Dako, Denmark) and finally counterstained with hematoxylin. The primary antibodies used were anti-BCAT1 (Proteintech, United States), anti-p-AKT (CST, United States), anti-CD31 (CST), anti-VEGF (CST), anti-Ki67 (CST), and anti-CD44 (CST). IHC staining of clinical samples was defined using four categories according to the percentage of positive cells: negative (0–25%), weakly positive (26–50%), positive (51–75%), and strongly positive (76–100%).

### Cell Lines and Culture

The human GC BGC823 cell line was obtained from American Type Culture Collection (Manassas, VA, United States). BGC823 cells were cultured in RIPM 1640 medium containing 10% fetal bovine serum (FBS, Gibco, United States), 1% (wt/vol) L-glutamine (Invitrogen, United States), penicillin (100 U/mL), and streptomycin (100 U/mL) at 37°C under 5% CO_2_. All cells were subcultured using 0.1% EDTA and 0.2% trypsin when confluent (>80% confluence).

### Lentiviral Production and Transduction

The psi-LVRU6GP lentiviral vector (GeneCopoeia, Inc., Guangzhou, China) containing a small hairpin RNA (shRNA) targeting BCAT1 (shRNA1#: 5′-GGCTAAAGAC CTAATAGTCAC-3′ or shRNA2#: 5′-GGAGAAACCTCATA TCAAGCC-3′) was used to silence BCAT1. The pEZ-Lv201 lentiviral vector (GeneCopoeia, Inc.) carrying a BCAT1 sequence (forward: 5′-GCGGTAGGCGTGTACGGT-3′, reverse: 5′-CTG GAATAGCTCAGAGGC-3′) was used to overexpress BCAT1. Recombinant lentiviruses were produced by transient transfection of HEK293T cells with the lentiviral vector by the calcium phosphate method. Recombinant lentivirus packaging was supported by FulenGen, Inc. (Guangzhou, China). The psi-LVRU6GP and pEZ-Lv105 lentiviral vectors were used as negative controls (NCs). For stable cell line generation, BGC823 cells were seeded in a six-well plate and cultured until they reached 30–40% confluence. Then, recombinant lentiviruses were transduced into BGC823 cells for 24 h. To produce stably transfected cell lines, transduced cells were screened in medium containing puromycin (2 μg/mL). Cells were incubated for another 2 weeks and harvested when the transduction efficiency was >90% as measured by the density of green fluorescent protein. Pooled clones were screened following the standard Western blot protocol.

### Colony Formation Assay

Transfected or treated BGC-823 cells were placed in six-well plates at a density of 500 cells/well and cultured for 2 weeks in a humidified incubator at 37°C with a 5% CO_2_ atmosphere. After removal of the culture medium, cells were fixed in methanol for 30 min and stained with trypan blue solution for another 30 min. Stained cells were washed with phosphate-buffered saline (PBS) three times, and colony numbers (>50 cells/colony) were counted under a light microscope (Nikon, Japan).

### Cell Proliferation Assay

Approximately 2 × 10^3^ transfected or treated BGC-823 cells at 70–80% confluence were cultured in a 96-well plate at 37°C for 24 h. Then, 10 μL thiazolyl blue tetrazolium blue (MTT) solution (5 mg/mL) was added to each well and cultured for another 4 h. After removal of the supernatant, 200 μL dimethyl sulfoxide was added to each well. Absorbance at 492 nm was detected using a microplate reader (Bio-Rad, United States).

### Flow Cytometry

The cell cycle assay was performed using flow cytometry. Transfected or treated BGC-823 cells were fixed in precooled 80% ethanol at −20°C overnight. Cell suspensions were centrifuged (4°C, 1,500 revolutions/min, 10 min) to collect the fixed cells. After washing with PBS, cells were stained with 0.5 mL FxCycle^TM^ PI/RNAse solution. Cell cycling was profiled using an Attune NxT flow cytometer in triplicate (Thermo Fisher Scientific, United States).

### Cell Invasion Assay

Transfected or treated BGC-823 cells were resuspended in serum-free medium. Matrigel-coated Transwell chambers were precooled at 4°C. Cell suspension (8 × 10^4^ cells/well) were added to the upper compartment of an 8-μm-pore Transwell chamber (Corning, United States) coated with Matrigel (BD Biosciences, United States). Complete medium containing 10% fetal bovine serum was used as a chemoattractant in the lower compartment. The Transwell chamber was maintained at 37°C for 24 h; then, non-invading cells were washed using PBS from the Matrigel upper surface. Invading cells were fixed with methanol for 30 min and stained with crystal violet for another 30 min. Stained cells were counted in five random fields per well under a light microscope (Nikon, Japan).

### Human Umbilical Vein Endothelial Cell (HUVEC) Tube Formation

Matrigel (50 μL; BD Biosciences) was plated in a precooled 96-well plate after thawing on ice and allowed to polymerize at 37°C in a humidified incubator with 5% CO_2_ for 30 min. HUVECs were removed from the complete medium, trypsinized, and resuspended in serum-free medium at a concentration of 1 × 10^6^ cells/mL. The cell suspension (20 μL) was mixed with 150 μL of the tumor-conditioned medium of transfected BGC-823 cells and then added to the Matrigel, followed by cultivation of 24 h. HUVECs after cocultivation were trypsinized, resuspended in serum-free medium at a concentration of 3 × 10^4^ cells/mL, and cultured in a Matrigel-coated 96-well plate for 24 h. Transfected BGC-823 cells were trypsinized and resuspended in DF12 medium at a concentration of 6 × 10^4^ cells/mL and cultured in a Matrigel-coated 96-well plate for 24 h. After cultivation, tube formation of HUVECs and BGC-823 cells was observed under an inverted microscope (Nikon, Japan); images were acquired from five sequential fields (×100 magnification).

### Western Blotting

Western blotting was performed using primary antibodies against BCAT1 (Proteintech), p-PI3K (CST), PI3K (CST), p-AKT (CST), AKT (CST), p-mTOR (CST), mTOR (CST), CD31 (CST), MMP-9 (CST), and VEGF (CST) according to the standard protocol. β-Actin (CST) was used an endogenous control. Protein bands were visualized with an enhanced chemiluminescence kit (Millipore, United States) and semiquantified subsequent to normalization to the density of the endogenous control using the Bandscan software.

### Immunofluorescence Assay

Cells were seeded in a 24-well plate at a density of 8 × 10^4^ and cultured to a confluence of 50%. After washing with serum-free medium, cells were fixed in 4% paraformaldehyde (15 min), washed three times with PBS containing 1% bovine serum albumin and 0.05% Tween-20, permeabilized with 0.02% TritonX-100 (5 min), and blocked with 10% serum (10 min). Then, cells were incubated with primary antibodies against CD31 (CST) and subsequently with secondary antibody. Nuclei were stained with DAPI. Images were obtained under a laser scanning confocal microscope (Leica Microsystems, Wetzlar, Germany).

### Mouse Xenograft Models and Treatment

A total of 20 specific-pathogen-free BALB/C nude mice (female, 4 weeks old) were purchased from Charles River Laboratories (Beijing, China). All animals were housed in a standard laboratory environment (25 ± 1°C, 60 ± 5% humidity, and 12-h light–dark cycle) with free access to food and water. Animal procedures were performed in accordance with National Institutes of Health Guide for the Care and Use of Laboratory Animals. The protocol was authorized by the institutional review board (NCC2017G-021).

Mice were randomly allocated to four groups (*n* = 5 per group) using a random-number table. Xenograft models were induced using BGC-823 cells with stable overexpression or knockdown of BCAT1. Briefly, BGC-823 cells with stably expressed BCAT1, or mock cells (3 × 10^6^), were, respectively, implanted into the forelimb axilla of BALB/C nude mice under sterile conditions. When tumors reached palpable size, their volumes were determined *in vivo* using an external caliper every 3–4 days. Meanwhile, LY294002 (25 mg/kg, MedChem Express, United States) was intraperitoneally injected into the xenograft models with BCAT1 overexpression or their NCs every 2 days. Tumor volumes were calculated by the following formula: *V* (mm^3^) = (length × width^2^)/2. After the 23-day cycle of treatment, all mice were killed by cervical dislocation in a fume cupboard, and tumors were harvested and weighed. After weighing, tumor tissues were collected for subsequent experiments, including hematoxylin-eosin (H&E) staining, Western blotting, and IHC staining according to standard protocols.

### GST Pull-Down and Mass Spectrometry Assays

GST (control) and GST-BCAT1 fusion proteins were expressed in BL21 (DE3) *Escherichia coli*; proteins were subsequently affinity purified and immobilized using glutathione sepharose 4B resin (GST resin, Cytiva, United States). GST resins bound with GST or GST-BCAT1 were incubated with the total protein (5 mg) extracted from GC cells at 4°C overnight. After washing of the resins, the bound proteins were eluted using an imidazole gradient, followed by visualization with sodium dodecyl sulfate polyacrylamide gel electrophoresis or Western blotting. The bands only present in GST-BCAT1 resin eluates were excised and subjected to mass spectrometry assay using an Agilent 1100 HPLC system connected to an LTQ Orbitrap Linear Ion Trap Mass Spectrometer. The fragment sequences were searched and analyzed using the Mascot database.

### Statistical Analysis

Statistical analysis was performed with SPSS statistical software (version 22.0, SPSS Inc., United States). Quantitative data were expressed as mean ± standard deviation, and qualitative data were expressed as frequencies and percentages. The statistical significance of differences between two groups was determined by Student’s *t*-test, and differences among multiple groups were analyzed by one-way analysis of variance with Tukey *post hoc* test. Multivariate analysis was performed with the Cox proportional hazards model. Correlation analysis was performed by Spearman rank correlation test. Statistical significance was set at a two-sided significance level of 0.05 with a 95% confidence interval.

## Results

### BCAT1 Is a Negative Prognostic Factor in Patients With GC

**FIGURE 1:**
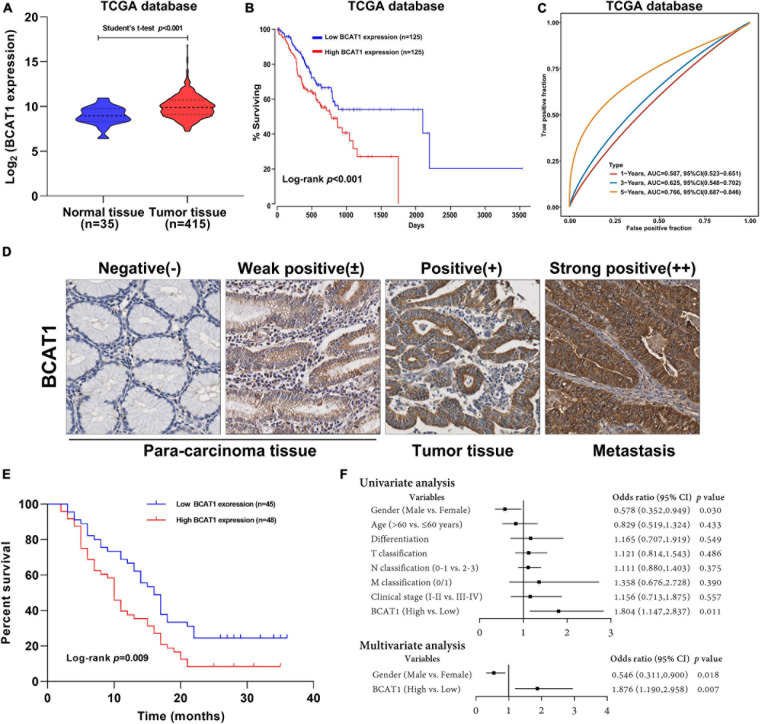
BCAT1 is a negative prognostic factor for patients with GC. **(A)** Expression of BCAT1 in tumor tissues and normal tissues from TCGA GC dataset. **(B)** Kaplan–Meier survival curve for OS in TCGA GC dataset, classified by BCAT1 expression (high: FPKM values above the 66th percentile; low: FPKM values below the 33rd percentile). **(C)** Time-dependent ROC analysis of BCAT1 in TCGA GC dataset. **(D)** Representative IHC images for the clinical samples from 95 GC patients **(E)** Kaplan–Meier survival curve of OS according to BCAT1 IHC staining in 95 GC patients (high: positive expression; low: negative expression). **(F)** Forest plot of the Cox regression analysis in 95 GC patients.

To investigate the potential role of BCAT1 in GC progression, we first analyzed the expression levels of BCAT1 in GC samples from TCGA. As shown in Figure 1A, BCAT1 was significantly upregulated in GC tissues. Kaplan–Meier survival analysis revealed that patients with higher BCAT1 expression had lower OS compared with those with lower BCAT1 expression (Figure 1B). Moreover, time-dependent ROC analysis showed that BCAT1 expression had satisfactory predictive accuracy for 5-year OS, with an area under the curve of 0.766 in patients with GC (Figure 1C). To verify these findings, BCAT1 expression was detected in clinical samples. IHC staining for BCAT1 expression was negative or weakly positive in paracarcinoma tissues but positive in tumor tissues and even strongly positive in tumors with metastasis (Figure 1D), implying that BCAT1 expression may be related to GC progression. Similar to the results from TCGA, patients with high BCAT1 expression exhibited lower OS compared with those with low BCAT1 expression (Figure 1E). In addition, the expression of BCAT1 was associated with T classification, N classification, and advanced clinical stage (Table 1). After adjusting for confounding factors, multivariate analysis further confirmed that patients with high BCAT1 expression had relatively high risk of low survival (Figure 1F). In summary, these results suggest that BCAT1 is frequently upregulated in GC and may predict poor prognosis in patients with GC.

**TABLE 1:** Relationship between BCAT1 expression and clinical parameters in 93 GC patients.

**Clinical parameters**	**Case (n)**	**BCAT1 expression (no,%)**		***p*-value**
		**Low (*n* = 45)**	**High (*n* = 48)**	
**Age (year)**				0.819
>60	30	14 (31.1)	16 (33.3)	
≤60	63	31 (68.9)	32 (66.7)	
**Gender**				0.672
Male	68	32 (71.1)	36 (75.0)	
Female	25	13 (28.9)	12 (25.0)	
**Differentiation**				0.102
Poor	78	35 (77.8)	44 (91.7)	
Moderate		9 (20.0)	4 (8.3)	
Well	15	1 (2.2)	0 (0.0)	
**T classification**				0.022
T1	1	1 (2.2)	0 (0.0)	
T2	29	20 (44.4)	9 (18.8)	
T3	47	19 (42.2)	28 (58.3)	
T4	16	5 (11.1)	11 (22.9)	
**N classification**				0.049
0–1	32	20 (44.4)	12 (25.0)	
2–3	61	25 (55.6)	36 (75.0)	
**M classification**				0.136
0	82	42 (93.3)	40 (83.3)	
1	11	3 (6.7)	8 (16.7)	
**Clinical stage**				0.007
I–II	27	19 (42.2)	8 (16.7)	
III–IV	66	26 (57.8)	40 (83.3)	

### BCAT1 Regulates Various Downstream Pathways in TCGA GC Dataset

**FIGURE 2:**
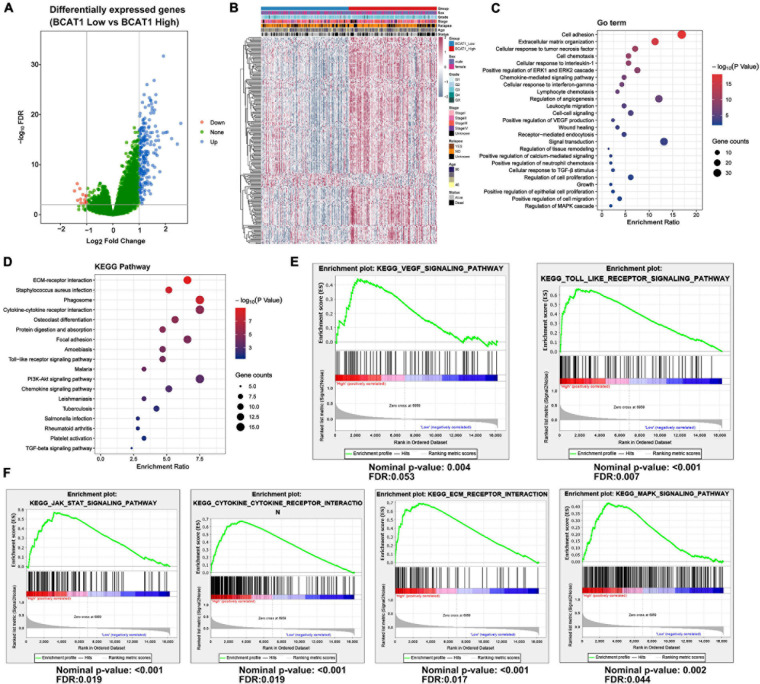
BCAT1 regulates various downstream pathways in TCGA GC data. **(A)** Volcano plot and **(B)** heatmap showing DEGs between BCAT1-high and BCAT1-low expression groups in the TCGA GC dataset. **(C)** GO analysis and **(D)** KEGG pathway analysis revealed the representative enrichment pathways. **(E)** GSEA plot showing the positive correlation between BCAT1 and angiogenesis-related pathways. **(F)** GSEA plot showing the positive correlation between BCAT1 and invasion- or proliferation-related pathways.

To further explore the function and role of BCAT1 in GC progression, an extensive bioinformatics analysis was performed using RNA-sequencing data from TCGA in cases of high and low BCAT1 expression. The BCAT1-induced oscillation gene patterns are shown in the volcano plot and heatmap in Figures 2A,B. A total of 217 genes showed significantly different expression between cases with high vs. low BCAT1 expression, of which 204 were upregulated and 13 were downregulated. GO enrichment analysis revealed that these DEGs were involved in the regulation of cell adhesion, proliferation, growth, migration, angiogenesis, and VEGF production (Figure 2C), implying the participation of BCAT1 in GC progression. KEGG pathway analysis also demonstrated that these DEGs were enriched in several important pathways, including extracellular matrix (ECM)–receptor interaction, cytokine–cytokine receptor interaction, focal adhesion, Toll-like receptor signaling pathway, and PI3K–Akt signaling pathway (Figure 2D), which are all crucial for tumorigenesis and angiogenesis. Furthermore, we performed GSEA to verify the correlations between BCAT1 and these functional pathways. As shown in Figure 2E, BCAT1 was positively correlated with angiogenesis-related pathways, including the VEGF signaling pathway and Toll-like receptor signaling pathway. Figure 2F shows the positive correlations between BCAT1 and invasion- or proliferation-related pathways, including the JAK–STAT and MAPK signaling pathways. We next confirmed these results by *in vitro* experiments.

### BCAT1 Promotes Proliferation, Invasion, and Angiogenesis of GC Cells *in vitro*

**FIGURE 3:**
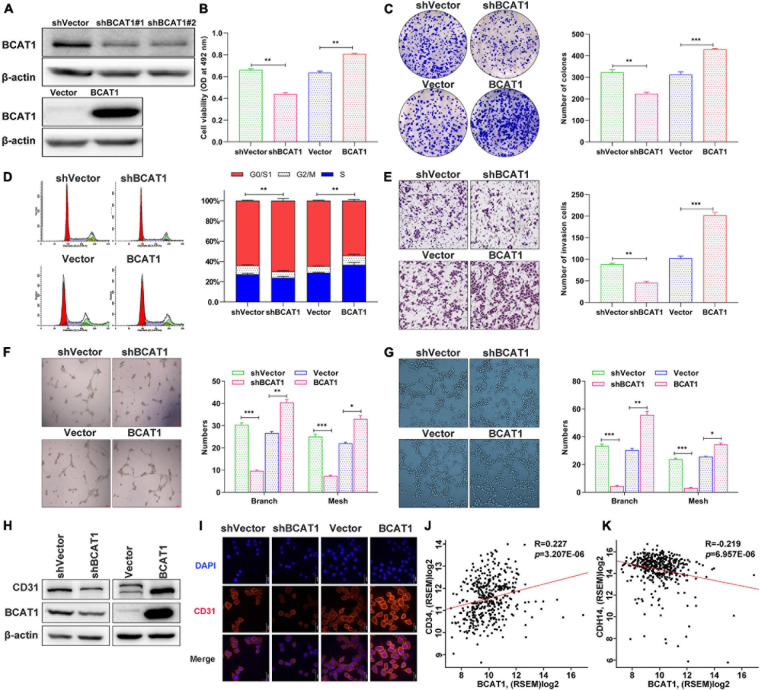
BCAT1 promotes proliferation, invasion, and angiogenesis of GC cells *in vitro*. BGC823 cells were transfected with lentivirus vector to induce BCAT1 silencing (shBCAT1) or overexpression (BCAT1). **(A)** Western blot confirming the downregulation (upper panel) or upregulation (lower panel) of BCAT1 in BGC823 cells. **(B)** MTT assay showing the viability of transfected cells. **(C)** Representative images of colony formation assay in transfected cells (right panel) and quantitative results (left panel). **(D)** Representative images of flow cytometric cell cycle analysis in transfected cells (right panel) and quantitative results (left panel). **(E)** Representative images of Transwell invasion assay in transfected cells (right panel) and quantitative results (left panel). **(F,G)** HUVEC tube formation assays were performed to evaluate angiogenesis ability. Representative images and quantitative results of HUVECs **(F)** and BGC-823 cells **(G)** are shown. **(H)** Western blot and **(I)** immunofluorescence assay for CD31 protein expression in transfected cells. **(J,K)** Correlation analysis of BCAY1 expression with CD34 angiogenic marker, **(J)** or CDH14 invasion marker, **(K)** in TCGA GC dataset. *p < 0.05, **p < 0.01, ***p < 0.001. Data are expressed as mean ± SEM of three independent experiments.

To verify the effects of BCAT1 on cell proliferation, invasion, and angiogenesis, we induced stable BCAT1 silencing (shBCAT1) or overexpression (BCAT1) in BGC823 cells. The successful silencing or overexpression of BCAT1 was confirmed by Western blotting (Figure 3A). The shBCAT1#1-transfected cells were used for subsequent experiments. The MTT assay showed that cell viability was significantly decreased by BCAT1 silencing but enhanced by BCAT1 overexpression (Figure 3B). Similarly, the colony formation assay also showed a decrease in proliferation with BCAT1 silencing and an increase in proliferation with BCAT1 overexpression (Figure 3C). Flow cytometric cell cycle analysis demonstrated that BCAT1 silencing blocked the cell cycle in G0/G1 to S phase, whereas BCAT1 overexpression promoted these phases of the cell cycle (Figure 3D). Moreover, invasion ability was suppressed in BCAT1-silenced cells but enhanced in BCAT1-overexpressing cells (Figure 3E). A HUVEC tube formation assay was conducted to examine whether BCAT1 affected angiogenesis. As shown in Figure 3F, the numbers of branches and meshes of HUVECs were obviously reduced in the shBCAT1 group, whereas they were increased in the BCAT1 group, compared with the corresponding NC. Consistent results were observed for the numbers of branches and meshes of BGC-823 cells (Figure 3G). We also detected the expression of CD31 (a tumor angiogenic marker). Both Western blotting and immunofluorescence staining verified the downregulation of CD31 by BCAT1 silencing and its downregulation by BCAT1 overexpression (Figures 3H,I). Supplementary analysis using the TCGA GC dataset also showed that BCAT1 was positively correlated with CD34 (an angiogenic marker, Figure 3J) and negatively correlated with CDH14 (an invasion marker, Figure 3K). Collectively, these results indicate that BCAT1 promotes cell proliferation, invasion, and angiogenesis.

### BCAT1 Activates PI3K/AKT/mTOR Signaling to Promote Proliferation and Angiogenesis of GC Cells *in vitro*

**FIGURE 4:**
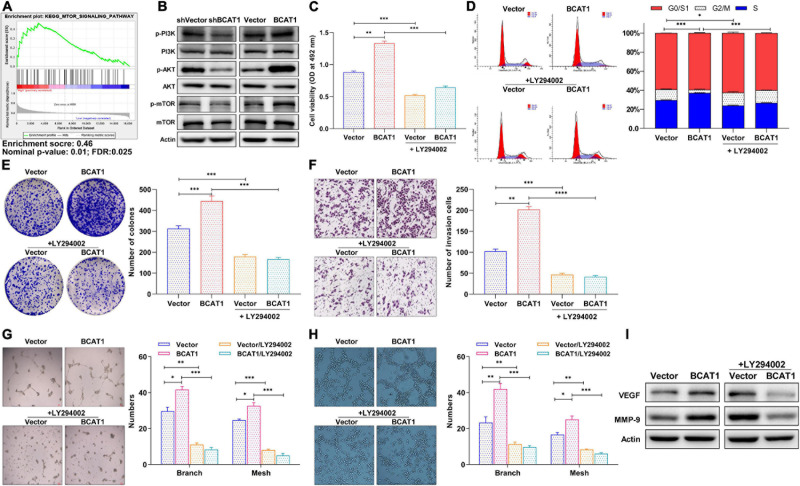
BCAT1 activates PI3K/AKT/mTOR signaling to promote proliferation and angiogenesis of GC cells *in vitro.* **(A)** GSEA plot showing positive correlation between BCAT1 and mTOR pathways. **(B)** Western blot showing expression of proteins in PI3K/AKT/mTOR signaling in transfected cells. BCAT1-overexpressing cells or NC cells were treated with LY294002 (10 μM). **(C)** MTT assay, **(D)** flow cytometric analysis, **(E)** colony formation assay, **(F)** Transwell invasion assay, and **(G,H)** HUVEC tube formation assay were performed to detect proliferation, cell cycle, invasion, and angiogenesis ability after LY294002 treatment. **(I)** Western blot for MMP-9 (invasion marker) and VEGF (angiogenesis marker) after LY294002 treatment. *p < 0.05, **p < 0.01, ***p < 0.001. Data are expressed as mean ± SEM of three independent experiments.

The GSEA plot obtained through bioinformatics analysis in the TCGA GC dataset showed that BCAT1 was positively correlated with the mTOR signaling pathway (Figure 4A). Thus, based on the KEGG and GSEA results, we subsequently examined the potential mechanism underlying the role of BCAT1-mediated PI3K/AKT/mTOR signaling in GC progression. Western blotting confirmed the activation of PI3K, AKT, and mTOR by BCAT1 overexpression, as well as the inhibition of PI3K/AKT/mTOR signaling by BCAT1 silencing (Figure 4B). Next, the BCAT1-transfected cells were exposed to a PI3K/AKT/mTOR signaling inhibitor, LY294002 (10 μM). The results showed that LY294002 blocked the BCAT1-induced enhancement of cell viability (Figure 4C), cell cycle progression (Figure 4D), colony formation (Figure 4E), and invasion (Figure 4F). Importantly, the inhibition of PI3K/AKT/mTOR signaling also reversed the BCAT1-induced increases in numbers of branches and meshes of both HUVECs and BGC-823 cells (Figures 4G,H). Furthermore, Figure 4I showed that the addition of LY294002 reversed the BCAT1-induced upregulation of MMP-9 (invasion marker) and VEGF (angiogenesis marker), supporting the results from phenotypic experiments. In summary, these data suggest that BCAT1 promotes GC progression by activating PI3K/AKT/mTOR signaling.

### BCAT1 Enhances Angiogenesis and Tumorigenicity by Activating PI3K/AKT/mTOR Signaling in GC Xenograft Models

**FIGURE 5:**
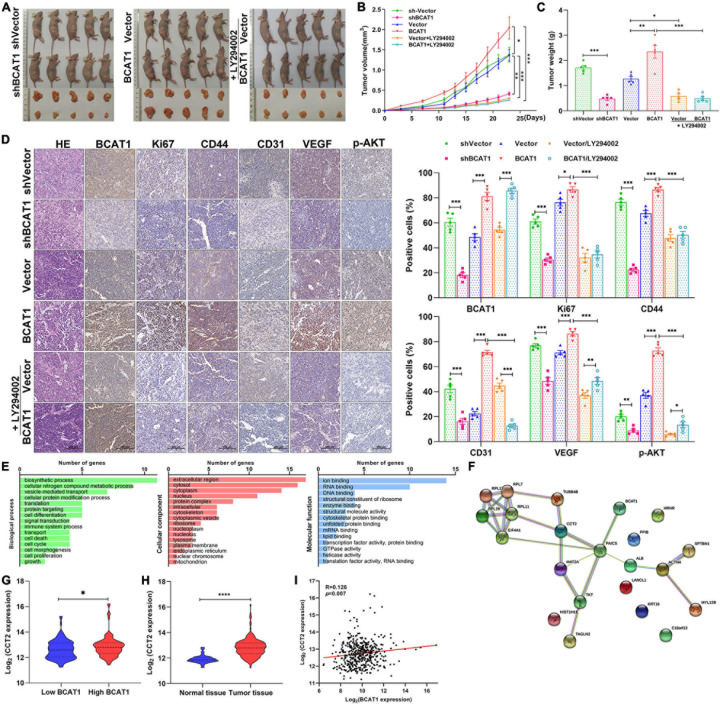
BCAT1 enhances angiogenesis and tumorigenicity by activating PI3K/AKT/mTOR signaling in GC xenograft models. **(A)** Tumor xenografts were generated using BCAT1-silencing or BCAT1-overexpressing BGC-823 cells. Mice with BCAT1 overexpression were treated with or without LY294002. Images of tumors are shown (*n* = 5). **(B)** Tumor growth curve and **(C)** tumor weight in each group. **(D)** Representative images of H&E staining and IHC staining for BCAT1, Ki67, CD44, CD31, and p-AKT (*n* = 5). **(E–I)** Analysis of BCAT1-interacting proteins in the exploratory research. **(E)** Representative enrichment pathways of 21 genes revealed by gene ontology (GO) analysis. **(F)** Network of 21 genes using String-DB analysis. **(G)** CCT2 was differentially expressed between BCAT1-high and -low group (top quartile vs. bottom quartile in TCGA database). **(H)** CCT2 expression in The Cancer Genome Atlas (TCGA) gastric cancer dataset. **(I)** Correlation between BACT1 and CCT2 expression in TCGA gastric cancer dataset. ****p < 0.0001.

To further confirm the tumorigenicity of BCAT1 *in vivo*, xenograft models with BCAT1 overexpression and silencing were established. In addition, LY294002 was intraperitoneally injected into BCAT1 overexpression models to verify the relationship between BCAT1 and PI3K/AKT/mTOR signaling *in vivo*. Tumors derived from BCAT1-silenced cells showed an obvious reduction in volume compared with tumors from sh-vector cells (Figures 5A,B). We also observed that tumors derived from BCAT1-overexpressing cells showed increased growth compared with tumors derived from vector cells; this phenomenon was reversed by the addition of LY294002 (Figures 5A,B). Similar results for tumor weights were found at day 23 (Figure 5C). Furthermore, H&E staining revealed that tumorigenicity was inhibited by BCAT1 silencing but enhanced by BCAT1 overexpression (Figure 5D). In addition, we detected the expression of proteins related to proliferation, angiogenesis, and signaling by IHC (Figure 5D). The results demonstrated that BCAT1 silencing markedly suppressed proliferation (Ki67), invasion (CD44), and angiogenesis (CD31 and VEGF), as well as the activation of PI3K/AKT/mTOR signaling (p-AKT). Conversely, BCAT1 overexpression induced an obvious upregulation of Ki67, CD44, CD31, VEGF, and p-AKT compared with controls. Moreover, the BCAT1-induced upregulations were completely reversed by LY294002. Taken together, these results indicate that BCAT1 enhances angiogenesis and tumorigenicity by activating PI3K/AKT/mTOR signaling in GC xenograft models, further supporting the findings of the bioinformatics analysis and *in vitro* experiments.

As exploratory research, we attempted to identify the BCAT1-interacting proteins by GST pull-down and mass spectrometry assays. Total proteins were extracted from GC cells BGC823 and pulled down by GST resin bound with GST (control) or GST-BCAT1. After identification by mass spectrometry assays, a total of 21 BCAT1-interacting proteins were obtained (Supplementary Table 1). GO analysis showed that these genes were significantly enriched in gene sets involved in cell differentiation, death, cycling, proliferation, extracellular region, and ion binding (Figure 5E), further supporting the tumorigenicity of BCAT1 in GC. In TCGA, String-DB analysis showed an indirect binding relationship between BCAT1 and CCT2, implying that BCAT1 may interact with CCT2 in GC progression (Figure 5F). Moreover, CCT2 was found to be differentially expressed between BCAT1-high and -low group (Figure 5G). Further analysis in TCGA demonstrated that CCT2 was upregulated in tumor tissues compared with normal tissues (Figure 5H) and positively correlated with BCAT1 expression (Figure 5I). Overall, we speculated that BCAT1 may interact with CCT2 to promote GC progression. However, the detailed mechanism remains to be investigated.

## Discussion

**FIGURE 6:**
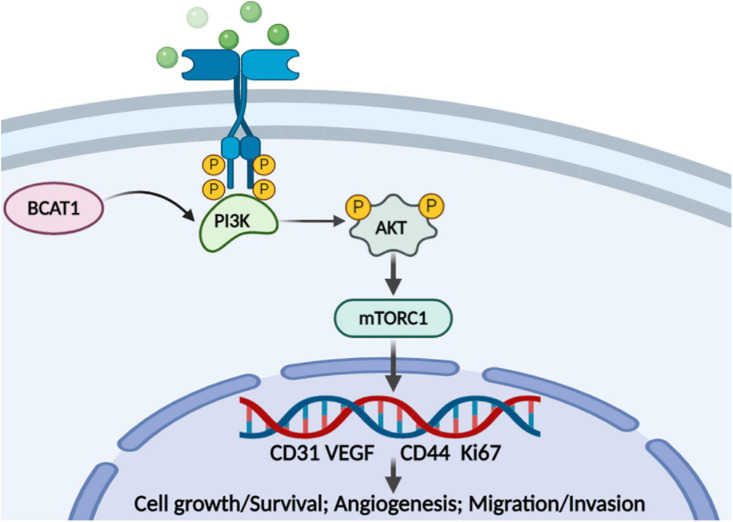
Proposed model for the role of BCAT1/PI3K/AKT/mTOR axis in regulating GC angiogenesis and tumorigenicity.

In recent years, antiangiogenesis has become a promising strategy for tumor therapy (Ferrara and Kerbel, 2005). Notably, GC is a highly angiogenic cancer with a high mortality rate. Nevertheless, most antiangiogenic agents have been reported to show no benefit to OS compared with chemotherapy alone in local or advanced GC (Hsieh and Tsai, 2019). The present study aimed to provide insights into the targets associated with tumor progression in GC, especially those related to angiogenesis. To the best of our knowledge, the present study is the first to explore the function of BACT1 in GC progression by bioinformatics analysis and *in vitro* and *in vivo* experiments. We confirmed the tumorigenicity of BCAT1 in GC progression and, more importantly, proposed a potential mechanism by which BCAT1 contributes to angiogenesis and malignant phenotypes by activating the PI3K/AKT/mTOR pathway (Figure 6).

BCAT1-mediated BACC catabolism is a conserved regulator of diverse physiological and pathological processes, including tumor development (Zhang and Han, 2017). Numerous studies have revealed that BCAT1 is overexpressed in several malignancies; more importantly, it is in involved in various malignant phenotypes, including changes in cell proliferation, cell cycle progression, differentiation, and apoptosis (Wang et al., 2015). Here, we verified the higher expression of BCAT1 in GC tumor tissues compared with normal tissues in both TCGA data and clinical samples, similar to the results of previous studies (Zhou et al., 2013; Chang et al., 2016). In addition, the bioinformatics analysis, including gene expression profiling as well as the associated GO and KEGG analyses and GSEA, showed that BCAT1 might affect invasion- and proliferation-related pathways, including ECM–receptor interaction, cytokine–cytokine receptor interaction, focal adhesion, and the PI3K–Akt signaling pathway, which all have critical roles in mediating survival signals in cancer (Martini et al., 2014; Bao et al., 2019). Thus, these results implied a possible mechanistic link between BCAT1 and the regulation of cell adhesion, proliferation, and growth in GC progression. Then, we confirmed the effects of BCAT1 on cell proliferation and invasion. As expected, we found that BCAT1 promoted cell viability, colony formation, and cell invasion and decreased G1/G0-phase cell cycle arrest. Certainly, we acknowledged that the present results came from only one GC cell line, which may weaken the strength of our conclusion. Notably, we investigated the role of BCAT1 from multiple aspects, including the comprehensive bioinformatics analysis, and even the *in vivo* experiments, which could also verify the tumorigenicity of BCAT1. Additionally, our results were compatible with those of previous studies on various tumors, including GC, gliomas, lung cancer, and breast cancer (Tönjes et al., 2013; Zhang and Han, 2017; Lin et al., 2020; Wang et al., 2020), which provided additional evidence that supported our finding. Overall, in the present study, we achieved a comprehensive understanding of BCAT1 tumorigenicity, in which the overexpression of BCAT1 promoted cell proliferation and invasion in GC progression.

Angiogenesis is emerging as an attractive target in cancer therapy, as it not only delivers oxygen and nutrients to tumor cells but can also provide a route for metastatic spread (Attoub et al., 2015). The most important finding of the present study is the illustration of BCAT1’s antiangiogenic property. First, functional analyses implicated BCAT1 in the regulation of angiogenesis. Moreover, the results of KEGG pathway analysis and GSEA further implied that BCAT1 was positively associated with PI3K/AKT/mTOR signaling and Toll-like receptor signaling, as well as the more critical VEGF signaling pathway, which are all pivotal in angiogenesis. As previously reported, the activation of PI3K/AKT/mTOR signaling induces VEGF secretion by hypoxia-inducible factor 1 (HIF-1)–dependent and HIF-1–independent mechanisms and also regulates the expression of other angiogenic factors such as nitric oxide and angiopoietins (Karar and Maity, 2011). Besides, Toll-like receptor signaling has been reported to contribute to angiogenesis through increasing VEGF production (Belmont et al., 2014). It is well recognized that VEGF and HIF-1 are potent driver of angiogenesis in human tumors (Jiang and Liu, 2008). In accordance with these findings, we confirmed the upregulation of VEGF by BCAT1 overexpression in xenograft models. By HUVEC tube formation assays, we further confirmed the promotion by BCAT1 of the tube formation abilities of both HUVECs and GC cells; this is indicative of sprouting angiogenesis (Ponce, 2009). Besides, CD31 and CD34, which are commonly used as angiogenic markers, were upregulated by BCAT1 overexpression, further suggesting that BCAT1 facilitates angiogenesis in GC. Similar to our findings, Cho et al. demonstrated that BCAT1 silencing inhibited the expression of CD34 and HIF-1α, proving the antiangiogenic role of BCAT1 in patients with glioblastoma (Cho et al., 2016). The above evidence suggests that BCAT1 may have the potential to serve as a potent antiangiogenic target in the treatment of GC.

Although functions of BCAT1 in various tumors have been reported, there is limited evidence regarding its downstream pathways. Here, GSEA revealed several signaling pathways involving the expression of BCAT1, including the mTOR pathway, JAK–STAT pathway, and cytokine–receptor interactions. The PI3K/AKT/mTOR pathway has been the focus of much research attention as a key potential target in numerous cellular functions, including proliferation, adhesion, migration, invasion, metabolism, and survival (Slomovitz and Coleman, 2012). Moreover, a previous review has demonstrated that activation of the PI3K/AKT/mTOR pathway in tumor cells can increase levels of VEGF, nitric oxide, and angiopoietins to regulate angiogenesis (Karar and Maity, 2011). Therefore, we mainly focused on the effects of BCAT1 on the PI3K/AKT/mTOR pathway. As expected, the activation of PI3K, AKT, and mTOR induced by BCAT1 overexpression were confirmed by Western blotting and IHC both *in vitro* and *in vivo*. Activation of the PI3K/AKT/mTOR pathway has been shown to promote growth, proliferation, and survival in cancer (LoRusso, 2016). More importantly, LY294002, an inhibitor of the PI3K/AKT/mTOR pathway, markedly reversed BCAT1-mediated tumorigenicity, including the phosphorylation of AKT; increased cell viability, proliferation, invasion, and angiogenesis; and tumor growth. These results suggest that the PI3K/AKT/mTOR pathway is a downstream pathway regulated by BCAT1. BCAT1 is a cytoplasmic enzyme that can catalyze the transamination of the BCAAs, including leucine, valine, and isoleucine, to their respective α-ketoacids and glutamate (Neinast et al., 2019). BCAAs, particularly leucine, are crucial signaling molecules in the PI3K/AKT/mTOR pathway (Song et al., 2020). There is evidence that BCAAs as potent nutrient signals together with growth factors regulate the activation of the PI3K/Akt/mTOR signaling network (Hassan et al., 2013). These amino acids can selectively activate mTOR complex 1 (mTORC1) or mTOR complex 2 (mTORC2) under different situations (Tato et al., 2011). As Tato et al. reported, amino acids could induce a rapid phosphorylation of AKT at both Thr-308 and Ser-473, which is mediated by the activity of class I PI3K, eventually activating mTORC2. Besides, amino acids could also activate mTORC1 via class III PI3K (Tato et al., 2011). Accordingly, we speculated that high expression of BCAT1 leads to the production of key amino acids, which further activate the PI3K/AKT/mTOR pathway, as reported by previous studies (Zhenyukh et al., 2017; Nie et al., 2018). Therefore, based on the results of GSEA and *in vitro* and *in vivo* experiments, our present study verified that BCAT1 activated the PI3K/AKT/mTOR pathway in GC progression. Accordingly, we speculated that GC patients with high BCAT1 expression may benefit more from treatment with inhibitors of the PI3K/AKT/mTOR pathway, such as everolimus, temsirolimus, buparlisib, and perifosine, which have been approved for anticancer therapy or are being tested in early clinical trials (Polivka and Janku, 2014; Singh et al., 2015). Thus, these findings may provide novel insights that could be used to improve the clinical efficacy of these inhibitors. This remains to be investigated in clinical trials.

Moreover, we identified that BCAT1 may interact with CCT2 to play a part in carcinogenesis. Here, we preliminarily found upregulation of CCT2 in the TCGA GC dataset and its positive correlation with BCAT1. Consistent with our results, CCT2 has been observed to be overexpressed and essential for the survival of various tumors, including liver, prostate, lung, and breast cancer (Guest et al., 2015; Carr et al., 2017). However, the interaction of BCAT1 with CCT2 has not been adequately verified; therefore, comprehensive research using techniques such as coimmunoprecipitation and hybridization tests is needed to confirm this interaction.

## Conclusion

In conclusion, the present study confirmed the overexpression of BCAT1 in GC. More importantly, we demonstrated that BCAT1 facilitated proliferation, invasion, and angiogenesis through the activation of the PI3K/AKT/mTOR pathway, thereby acting as an oncogene in GC progression. These findings indicate a potent therapeutic target for GC therapy, which could aid the optimization of antiangiogenesis strategies.

## Data Availability Statement

The original contributions presented in the study are included in the article/Supplementary Material, further inquiries can be directed to the corresponding author/s.

## Ethics Statement

The studies involving human participants were reviewed and approved by the Cancer Hospital, Chinese Academy of Medical Sciences and Peking Union Medical College. The patients/participants provided their written informed consent to participate in this study. The animal study was reviewed and approved by Cancer Hospital, Chinese Academy of Medical Sciences and Peking Union Medical College.

## Author Contributions

Y-LR and Y-MS: conceptualization and methodology. LY and JL: material preparation. L-XS and Z-HY: administrative support. XS and P-PZ: formal analysis and data collection and writing—original draft preparation. XS and L-CS: data analysis and interpretation. L-XS and LY: writing—review and editing. All authors read and approved the final manuscript.

## Conflict of Interest

The authors declare that the research was conducted in the absence of any commercial or financial relationships that could be construed as a potential conflict of interest.

## Footnotes

1 https://www.bioconductor.org/packages/release/bioc/html/TCGAbiolinks.html
