# Supplementary material for: BCAT1 Activates PI3K/AKT/mTOR Pathway and Contributes to the Angiogenesis and Tumorigenicity of Gastric Cancer
Source: Front Cell Dev Biol. 2021 Jun 7;9:659260. doi: 10.3389/fcell.2021.659260 (PMC8215359; doi:10.3389/fcell.2021.659260)
Supplement: Supplementary file 1 [file Data_Sheet_1.docx]

**Supplementary Table 1. The BCAT1-interacting proteins identified by GST-pull down and mass spectrometry assays**

| Code | Proteins | Note/Biological function |
| --- | --- | --- |
| 1 | BCAT1 | Branched chain amino-acid transaminase 1, cytosolic; Catalyzes the first reaction in the catabolism of the essential branched chain amino acids leucine, isoleucine, and valine |
| 2 | MYL12B | Myosin, light chain 12B, regulatory; Myosin regulatory subunit that plays an important role in regulation of both smooth muscle and nonmuscle cell contractile activity via its phosphorylation. Ph [...] |
| 3 | KRT16 | Keratin 16; Epidermis-specific type I keratin that plays a key role in skin. Acts as a regulator of innate immunity in response to skin barrier breach: required for some inflammatory checkpoint f [...] |
| 4 | SPTBN1 | Spectrin, beta, non-erythrocytic 1; Fodrin, which seems to be involved in secretion, interacts with calmodulin in a calcium-dependent manner and is thus candidate for the calcium-dependent moveme [...] |
| 5 | LANCL1 | LanC lantibiotic synthetase component C-like 1 (bacterial); May play a role in EPS8 signaling. Binds glutathione |
| 6 | C16orf13 | Chromosome 16 open reading frame 13 |
| 7 | RPL13 | Ribosomal protein L13 |
| 8 | RPL7 | Ribosomal protein L7; Binds to G-rich structures in 28S rRNA and in mRNAs. Plays a regulatory role in the translation apparatus; inhibits cell-free translation of mRNAs |
| 9 | CCT2 | Chaperonin containing TCP1, subunit 2 (beta); Molecular chaperone; assists the folding of proteins upon ATP hydrolysis. As part of the BBS/CCT complex may play a role in the assembly of BBSome, a [...] |
| 10 | TKT | Transketolase; Catalyzes the transfer of a two-carbon ketol group from a ketose donor to an aldose acceptor, via a covalent intermediate with the cofactor thiamine pyrophosphate |
| 11 | RPL29 | Ribosomal protein L29 |
| 12 | HIST1H1E | Histone cluster 1, H1e; Histone H1 protein binds to linker DNA between nucleosomes forming the macromolecular structure known as the chromatin fiber. Histones H1 are necessary for the condensatio [...] |
| 13 | TAGLN2 | Transgelin 2 |
| 14 | PPIB | Peptidylprolyl isomerase B (cyclophilin B); PPIases accelerate the folding of proteins. It catalyzes the cis-trans isomerization of proline imidic peptide bonds in oligopeptides |
| 15 | ACTN4 | Actinin, alpha 4; F-actin cross-linking protein which is thought to anchor actin to a variety of intracellular structures. This is a bundling protein (Probable). Probably involved in vesicular tr [...] |
| 16 | RPL11 | Ribosomal protein L11; Binds to 5S ribosomal RNA (By similarity). Required for rRNA maturation and formation of the 60S ribosomal subunits. Promotes nucleolar location of PML (By similarity) |
| 17 | TUBB4B | Tubulin, beta 4B class IVb; Tubulin is the major constituent of microtubules. It binds two moles of GTP, one at an exchangeable site on the beta chain and one at a non-exchangeable site on the al [...] |
| 18 | HRNR | Hornerin; Component of the epidermal cornified cell envelopes |
| 19 | MAT2A | Methionine adenosyltransferase II, alpha; Catalyzes the formation of S-adenosylmethionine from methionine and ATP |
| 20 | ALB | Albumin; Serum albumin, the main protein of plasma, has a good binding capacity for water, Ca(2+), Na(+), K(+), fatty acids, hormones, bilirubin and drugs. Its main function is the regulation of [...] |
| 21 | PAICS | Phosphoribosylaminoimidazole carboxylase, phosphoribosylaminoimidazole succinocarboxamide synthetase |
| 22 | EIF4A1 | Eukaryotic translation initiation factor 4A1; ATP-dependent RNA helicase which is a subunit of the eIF4F complex involved in cap recognition and is required for mRNA binding to ribosome. In the c [...] |
